# Construction of immune-related lncRNA signature to predict aggressiveness, immune landscape, and drug resistance of colon cancer

**DOI:** 10.1186/s12876-022-02200-5

**Published:** 2022-03-17

**Authors:** Yonggan Xue, Bobin Ning, Hongyi Liu, Baoqing Jia

**Affiliations:** Department of General Surgery, Chinese PLA General Hospital, No. 28, Fuxing Road, Haidian District, Beijing, 100853 People’s Republic of China

## Abstract

**Background:**

Colon cancer remains one of the most common malignancies across the world. Thus far, a biomarker, which can comprehensively predict the survival outcomes, clinical characteristics, and therapeutic sensitivity, is still lacking.

**Methods:**

We leveraged transcriptomic data of colon cancer from the existing datasets and constructed immune-related lncRNA (irlncRNA) pairs. After integrating with clinical survival data, we performed differential analysis and identified 11 irlncRNAs signature using Lasso regression analysis. We next plotted the 1-, 5-, and 10-year curve lines of receiver operating characteristics, calculated the areas under the curve, and recognized the optimal cutoff point. Then, we validated the pair-risk model in terms of the survival outcomes of the patients involved. Moreover, we tested the reliability of the model for predicting tumor aggressiveness and therapeutic susceptibility of colon cancer. Additionally, we reemployed the 11 of irlncRNAs involved in the pair-risk model to construct an expression-risk model to predict the prognostic outcomes of the patients involved.

**Results:**

We recognized a total of 377 differentially expressed irlncRNAs (DEirlcRNAs), including 28 low-expressed and 349 high-expressed irlncRNAs in colon cancer patients. After performing a univariant Cox analysis, we identified 115 risk irlncRNAs that were significantly correlated with survival outcomes of patients involved. By taking the overlap of the DEirlcRNAs and the risk irlncRNAs, we ultimately recognized 55 irlncRNAs as core irlncRNAs. Then, we established a Cox HR model (pair-risk model) as well as an expression HR model (exp-risk model) based on 11 of the 55 core irlncRNAs. We found that both of the two models significantly outperformed the commonly used clinical characteristics, including age, T, N, and M stages when predicting survival outcomes. Moreover, we validated the pair-risk model as a potential tool for studying the tumor microenvironment of colon cancer and drug susceptibility. Additionally, we noticed that combinational use of the pair-risk model and the exp-risk model yielded a more robust approach for predicting the survival outcomes of patients with colon cancer.

**Conclusions:**

We recognized 11 irlncRNAs and created a pair-risk model and an exp-risk model, which have the potential to predict clinical characteristics of colon cancer, either solely or conjointly.

**Supplementary Information:**

The online version contains supplementary material available at 10.1186/s12876-022-02200-5.

## Background

Although remarkable progress has been achieved in tackling colorectal cancer over the past decades, it remains the second in terms of mortality and the third in terms of incidence [1]. It is reported that colorectal cancer takes a toll on almost 700,000 people every year [2]. The global burden of colorectal cancer is projected to be 2.2 million new cases by 2030, with 1.1 million deaths from the disease [3]. It is well established that both genetic predisposition and environmental factors play a role in developing colorectal cancer [4]. Nearly a fifth of patients with colorectal cancer might have a positive family history of gene-related diseases like familial adenomatous polyposis and hereditary non-polyposis colorectal cancer [5]. Environmental factors, such as smoking, excessive alcohol consumption, and increased red meat intake, are mostly considered contributors to colorectal cancer [6]. While the highest rates of incidence are found in developed countries, the incidence of colorectal cancer appears to rapidly increase in developing nations due to more people shifting to Western dietary patterns and lifestyles [7].

Surgical resection of the tumor is still considered the cornerstone of curative-intent treatment. Benefiting from the improving surgical performance and equipment, some patients, especially those who are at early stages, can be cured without further interventions like radiotherapy and chemotherapy [8]. By contrast, for those who are diagnosed with a local advanced or distant metastatic tumor, systemic treatment, like adjuvant chemotherapy and immune therapy, should be considered or even prioritized [9]. However, the hard hurdle facing gastroenterologists and oncologists during the treatment of colorectal cancer is how to stratify patients who are suitable to a specific regimen. Therefore, it is urgently required a parameter to robustly predict the clinical characteristics and drug sensitivity of patients with colorectal cancer.

Long non-coding RNA (lncRNA), referred to as transcripts larger than 200 nucleotides, are transcribed by RNA polymerase II but not translated into proteins [10]. Biologically, lncRNA exerts a variety of functions at different levels of gene expression, including transcription, post-transcription, and chromatin modification [11]. When it comes to their role in cancer, lncRNA plays important roles in the process of tumorigenesis and cancer cell evolution via their influence on gene expression, immune response, and drug resistance [12]. For example, Carpenter et al. reported that LincRNA-Cox2 acts as an essential mediator regulating both activation and repression of a series of immune gene expression in macrophages [13]. Huang et al. found that lncRNA Morrbid subtly controls the life expectancy of neutrophils, eosinophils, and monocytes through regulating the expression of Bim in response to extracellular stimulation [14]. In the context of the tumor, Li et al. performed a comprehensive examination of the landscape of lncRNAs across 33 cancer types and found that the expression of lncRNAs tended to show a cancer-type specific pattern. Moreover, the perturbated transcription of lncRNA was significantly associated with the signature of immune cell infiltration [15].

However, the influence of irlncRNA expression profiles on colorectal cancer is rarely evaluated. In this study, the irlncRNA expression signature in colorectal cancer was analyzed from the TCGA dataset. After a series of analyses and validation processes, we eventually established an 11 irlncRNA pairs-based risk assessment model that is closely associated with tumor aggressiveness, survival, and drug resistance of colon cancer. Additionally, based on the same pool of the core irlncRNAs, we constructed an expression risk (exp-risk) model that is indicative of clinical traits and prognostic outcomes. Importantly, by integrating those two risk models, we could more robustly foresee the survival outcomes of patients with colon cancer.

## Methods

### Data download and processing

**Fig. 1:**
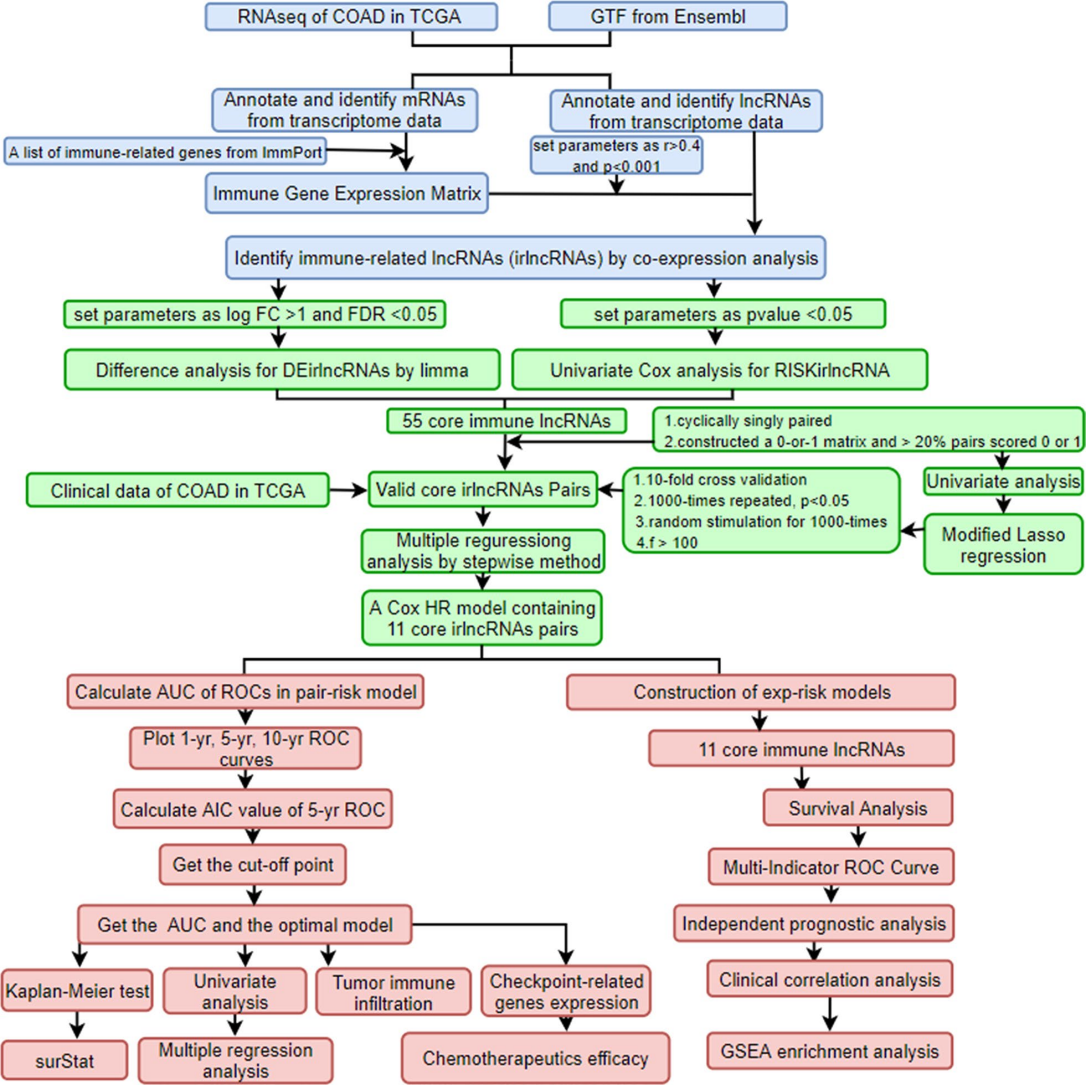
The process flow of the present study

The overall workflow is shown in Fig. 1. Briefly, after collecting transcriptional profiles, 473 tumor samples and 41 para-cancer normal samples with their clinical materials were downloaded from the TCGA-COAD dataset (https://portal.gdc.cancer.gov/). For subsequent analyses, the lncRNAs and mRNAs were distinguished into two separate matrices according to GTF files obtained from the Ensemble (http://asia.ensembl.org).

### Immune-related genes and irlncRNAs

A total of 2483 immune-related genes were identified from the IMMPORT (https://www.immport.org) and the corresponding expression matrix was extracted by Perl language. Then a co-expression analysis was conducted between the immune-related genes and the lncRNAs through the R package “limma”. The lncRNAs with the filtering conditions of Pearson correlation coefficient > 0.5 and *P* value < 0.001 were considered closely related to the immune-related genes and regarded as irlncRNAs.

### Differential and survival analysis of irlncRNAs and construction of irlncRNA pairs

Differential analysis of the irlncRNAs was conducted by “limma”. The thresholds were set as logFC > 2 and *P* value < 0.05. Focusing on the irlncRNAs, a univariate Cox analysis was conducted. The overlap between the DEirlncRNA and RISKirlncRNA was identified. R packages “pheatmap” and “survival” were utilized for the operation. To reduce the complex calculations caused by data correction and the relevant bias, the core irlncRNAs were circularly selected, and a 0-or-1 matrix was constructed. For each cycle, every two lncRNAs were selected and their expression levels were compared. Assuming C is defined as 1 if the expression level of irlncRNA A is higher than irlncRNA B, otherwise, C is defined as 0. The irlncRNA pair was regarded as a valid match only when the proportion of irlncRNA pairs with an expression of 0 or 1 accounted for more than 20% of the total pairs.

### Establishment of irlncRNA-pairs signature

After merging the irlncRNA pairs with clinical profiles, a uniCox survival analysis of irlncRNA pairs was performed using the R package “survival”. *P* value < 0.05 were considered as prognostically relevant. After obtaining the prognosis-related irlncRNA pairs, we further cross-validated the model and conducted a 1000-times-repeated Lasso regression analysis to filter the ultimate irlncRNA pairs without redundant information and construct the irlncRNA pairs in cross-validation by R package “glmnet”. Then the package “survminer” was used to construct the uni- and multi-Cox analysis to obtain the optimized risk formula.

### Validation of the model and clinical relevance

To verify the stability of the signature, we potted the 1-, 5-, and 10-year overall survival (OS) ROC curves using R package “survivalROC”, following which the best cutoff point based on the 5-year OS was identified using the riskScore formula: RiskScore $=\sum\nolimits_{i=1}^{k}\beta iSi$. The colon cancer patients involved were divided into high- and low-risk groups based on the cutoff that maximizes the area under the curve (AUC). To further visualize the risk model, we arranged the patients in terms of their risk values and presented their survival statuses with different color dots. Then we generated the 12-year K-M survival curves for the two groups of patients by R package “survival” and “survminer” to compare the survival differences. The package “survival” in R language was used to calculate the risk value, OS, and survival status of the individual patients, and *P* value < 0.05 was considered statistically significant. We then used the package “survivalROC” to input both the clinical information and the risk model to compare the accuracy of the risk model versus other clinical traits. Using R packages “limma” and “ggpubr”, we further revealed the correlation between the risk scores and clinical traits. Clinical traits were also charted between high- and low-risk groups by virtue of chi-square tests, and the results were shown in the clinical correlation heat map by R package “ComplexHeatmap”.

### Immune correlation analysis

To explore the correlation between TME and the pair-risk model, we evaluated the stromal and immune infiltrating cells among the colon cancer patients by seven acknowledged algorithms, including XCELL, TIMER, QUANTISEQ, MCPCOUNTER, EPIC, CIBERSORT-ABS, and CIBERSORT. R packages “limma”, “scales”, “ggplot2”, and “ggtext” were utilized to visualize these results. Then, a Wilcoxon signed-rank test was performed to analyze the differences between high- and low-risk groups by “limma” and “ggpubr” packages.

### Correlation analysis of immune genes

TGFB1 and LAG3 were typical immune checkpoints, which were well documented playing a critical role in immune therapy. The distinct expressions of them between the high- and low-risk groups were analyzed by “limma” and visualized by “ggpubr”. We labeled *** if the *P* value was less than 0.001, ** if the *P* value was less than 0.01, and * if the *P* value was less than 0.05.

### Drug sensitivity analysis

To identify the relationship between the pair-risk model and therapeutic efficacy, we performed a therapeutic response analysis of the colon cancer patients involved by calculating the IC50 which has a strong relationship with drug sensitivity. With the packages “limma”, “ggpubr”, “pRRophetic”, and “ggplot2”, drug sensitivities between the high- and low-risk groups were calculated by Wilcoxon signed-rank test and the statistically significant results were shown as box drawings.

### Expression risk model construction

After identifying the irlncRNAs in the context of colon cancer, we constructed an exp-risk model to obtain the risk values of each patient and divided them into high-risk and low-risk groups. By using “survival” and “survminer”, we investigated whether there were differences in survival outcomes between the two groups. We also drew the ROC curves to predict the accuracy of our models. Next, we used the “pheatmap” in R package to plot risk curves. To make our study more convincing, we combined pair-risk score and expression-risk score, and divided the patients into 4 subgroups according to their different risk values in the two groups, and used the “survminer” and “survival” packages for statistical analysis. The presentation of the survival of patients in each subgroup was performed using the “survminer” and “survival” packages.

### Independent prognostic analysis

Next, with the "survival" in R package, we performed an independent prognostic analysis. We compared this model with our patient's clinical traits to determine if the lncRNA-based model can be used as an independent prognostic indicator for colon cancer.

### GSEA enrichment analysis

Finally, the GSEA enrichment analysis was conducted in the lncRNA-pair model and exp-model to demonstrate the activated KEGG pathways between the high- and low-risk groups.

## Results

### Identification of differentially expressed irlncRNAs (DEirlncRNAs) and risk irlncRNA in colon cancer

**Fig. 2:**
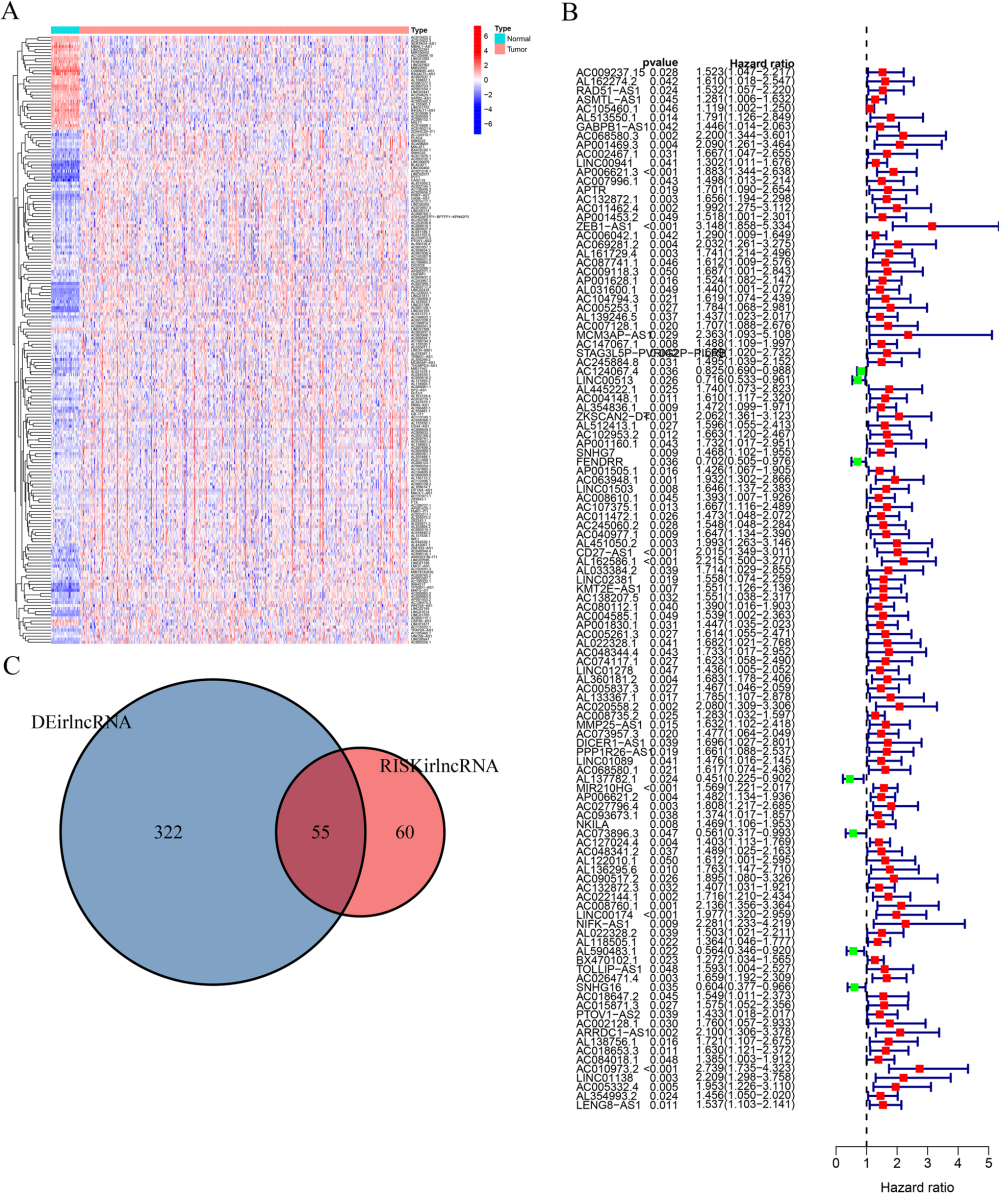
Identification of differentially expressed irlncRNAs. **A** The heatmap represents a total of irlncRNAs in colon cancer using TCGA datasets and annotated by Ensembl. **B** The forest map shows the relationship between DEirlncRNAs and the survival outcomes of patients with colon cancer. **C** The core irlncRNAs were determined by intersecting DEirlncRNA and RISKirlncRNA

Based on Pearson Correlation Analysis, 739 irlncRNAs were met on our selection criteria (Additional file 1: Table S1). Among them, 28 low-expressed and 349 high-expressed DEirlncRNAs were selected for further analysis (Fig. 2A). Then a univariant cox analysis identified 115 risk irlncRNAs that were significantly correlated with survival outcomes of patients with colon cancer (Fig. 2B, Additional file 2: Table S2). By taking the overlap of the DEirlcRNAs and the risk irlncRNAs, 55 irlncRNAs were recognized as core irlncRNAs (Fig. 2C, Additional file 3: Table S3). Furthermore, we constructed a Cox HR model (pair-risk model) as well as an expression HR model (exp-risk model) using a set of 11 core irlncRNAs (Tables 1, 2). Finally, we, separately, examined the relationship between the risk scores obtained from the two models and disease-related characteristics including clinical traits, survival statuses, TME, chemotherapeutic sensitivity, and signaling pathways.

**Table 1:** The risk model of immune lncRNA pairs

ID	coef	HR	HR.95L	HR.95H	*p* value
AC105460.1|AL590483.1	0.434869442	1.544761365	0.972868122	2.45283777	0.065275621
AP001469.3|AL137782.1	1.173749307	3.234095554	1.349471659	7.750717832	0.008487151
LINC00941|AL590483.1	0.412896035	1.511187907	0.968800939	2.357232325	0.068723513
AP001453.2|AC027796.4	− 0.773706228	0.461300212	0.284593551	0.747725607	0.001691186
AC007128.1|AL590483.1	0.704647609	2.023133626	1.276459379	3.206580434	0.002711144
AC124067.4|SNHG7	− 0.467395261	0.626632358	0.39248827	1.000458211	0.050224749
LINC00513|AC010973.2	− 0.801575511	0.448621599	0.279579346	0.719871986	0.000893026
LINC00513|LENG8-AS1	− 0.518086862	0.595659037	0.324938192	1.091929779	0.093828869
FENDRR|NKILA	− 0.781365548	0.457780463	0.299833856	0.698930252	0.000295668
AL451050.2|AL137782.1	0.544271131	1.723351826	1.046348925	2.838385406	0.032522231
AL137782.1|AL354993.2	− 0.554021701	0.57463415	0.367625261	0.898209241	0.015056368

**Table 2:** The risk model of immune lncRNA expression

ID	coef	HR	HR.95L	HR.95H	*p* value
AP001469.3	0.574505872	1.776252615	0.998051245	3.161233822	0.050780302
AP001453.2	− 0.363495071	0.695242154	0.432289518	1.118143358	0.133783345
AC007128.1	0.427241742	1.533023213	0.924508885	2.542063371	0.097768947
AC124067.4	− 0.269272158	0.763935316	0.611645819	0.954142331	0.017606639
LINC00513	− 0.419186774	0.657581365	0.470812319	0.918440818	0.013930483
FENDRR	− 0.310222378	0.733283872	0.517954308	1.038132571	0.080294969
AL137782.1	− 0.74476217	0.474847219	0.231960611	0.972061078	0.041601358
NKILA	0.514359624	1.672567087	1.217886075	2.296997001	0.001484107
AL590483.1	− 0.773741406	0.461283985	0.268857903	0.79143262	0.004966124
AC010973.2	1.34334251	3.831830054	2.112475134	6.950577227	9.80E−06
AL354993.2	0.422350358	1.525542918	0.972653578	2.392713343	0.065882942

### Construction of core irlncRNA pairs and establish of a pair-risk model

**Fig. 3:**
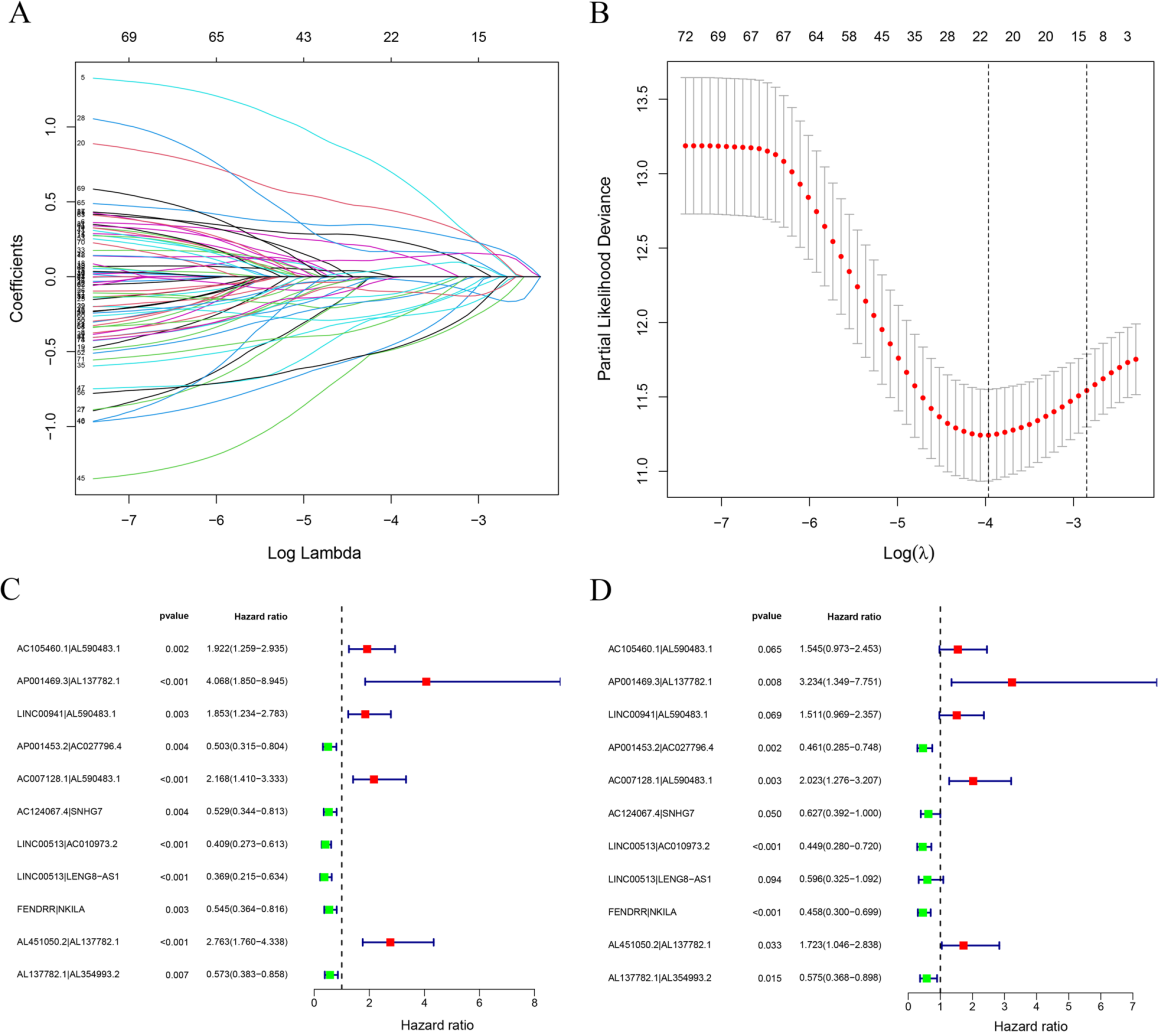
Construction of differentially expressed irlncRNA pairs. **A** Lasso coefficient profiles of the irlncRNAs were determined for establishing a pair-risk model. **B** The selection of the tuning parameter was performed via 10 times cross-validation in the Lasso model. **C**, **D** Forest maps show the clinical link of the 11 individual irlncRNA pairs identified by Cox proportional hazard regression to prognosis

To develop a model being adopted without considering the differences between the profiles, we paired those core irlncRNAs and then correlated them with the survival outcomes. With a threshold of *P* value < 0.05 in the univariate cox analysis, a total of 55 pairs of core irlncRNAs were determined to further construct the risk assessment model. After Lasso regression analysis of these 55 core irlncRNA pairs, 11 of them were finally selected for constructing the pair-risk model (Fig. 3A, B). We next performed univariate and multivariate Cox analyses of these 11 core irlncRNA pairs to corroborate their performance as independent clinical prognostic factors (Fig. 3C, D).

### Evaluation of the clinical predictivity of the pair-risk model

**Fig. 4:**
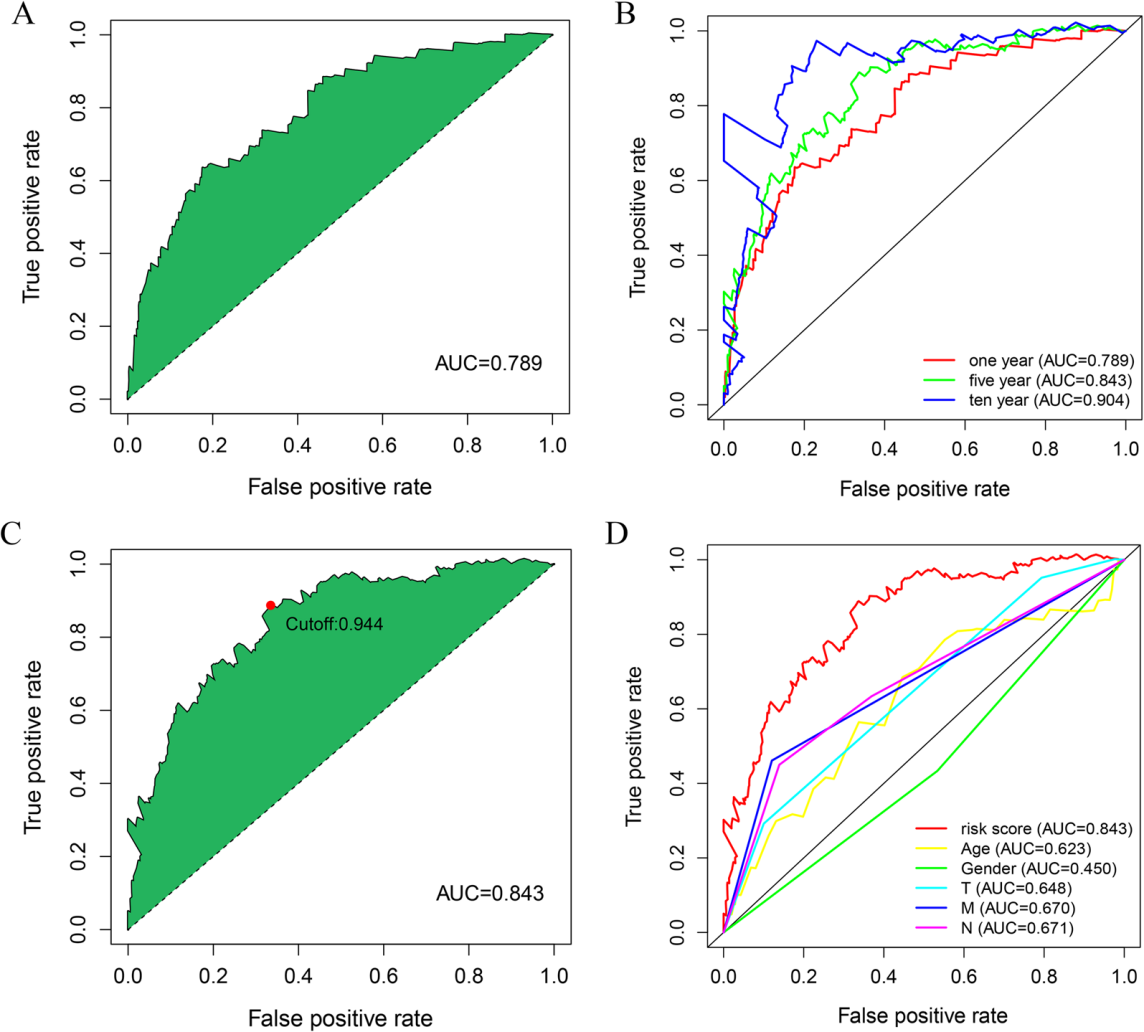
Establishment of a pair-risk assessment model by differentially expressed lncRNA pairs. **A** Plot shows the maximum AUC value of the model based on the core irlncRNA pairs. **B** The 1-, 5-, and 10-year ROC curves of the model suggest that all AUC values are over 0.70. **C** The maximum inflection point is recognized by the AIC. **D** Comparing risk score ROC curves with other common clinical characteristics indicate the superiority of this pair-risk model

We drew the 1-year ROC curve and found the area under the curve (AUC) was 0.789, indicating that the model can be employed with high accuracy to reflect the short-term overall survival outcomes of the patients involved (Fig. 4A). To further validate the stability and generalizability of the model, we plotted the 1-, 5-, and 10-year ROC curves (Fig. 4B). We noticed that the 5-year ROC curve was optimal with the best cutoff value of 0.843 (Fig. 4C). Based on this optimal cutoff value, we then categorized the colon cancer patients involved into two high- and low-risk groups. Of these, 237 patients were classified into the high-risk group, while the remaining 209 patients were allocated to the low-risk group. Next, by virtue of multi-metric ROC curves, we plotted the ROC curve of the risk model together with that of clinical characteristics including age, T, N, and M stages, in the identical diagram for comparison. The results showed that the AUC value of the risk model dramatically outperformed that of clinical parameters, indicative of the high performance of this model (Fig. 4D).

**Fig. 5:**
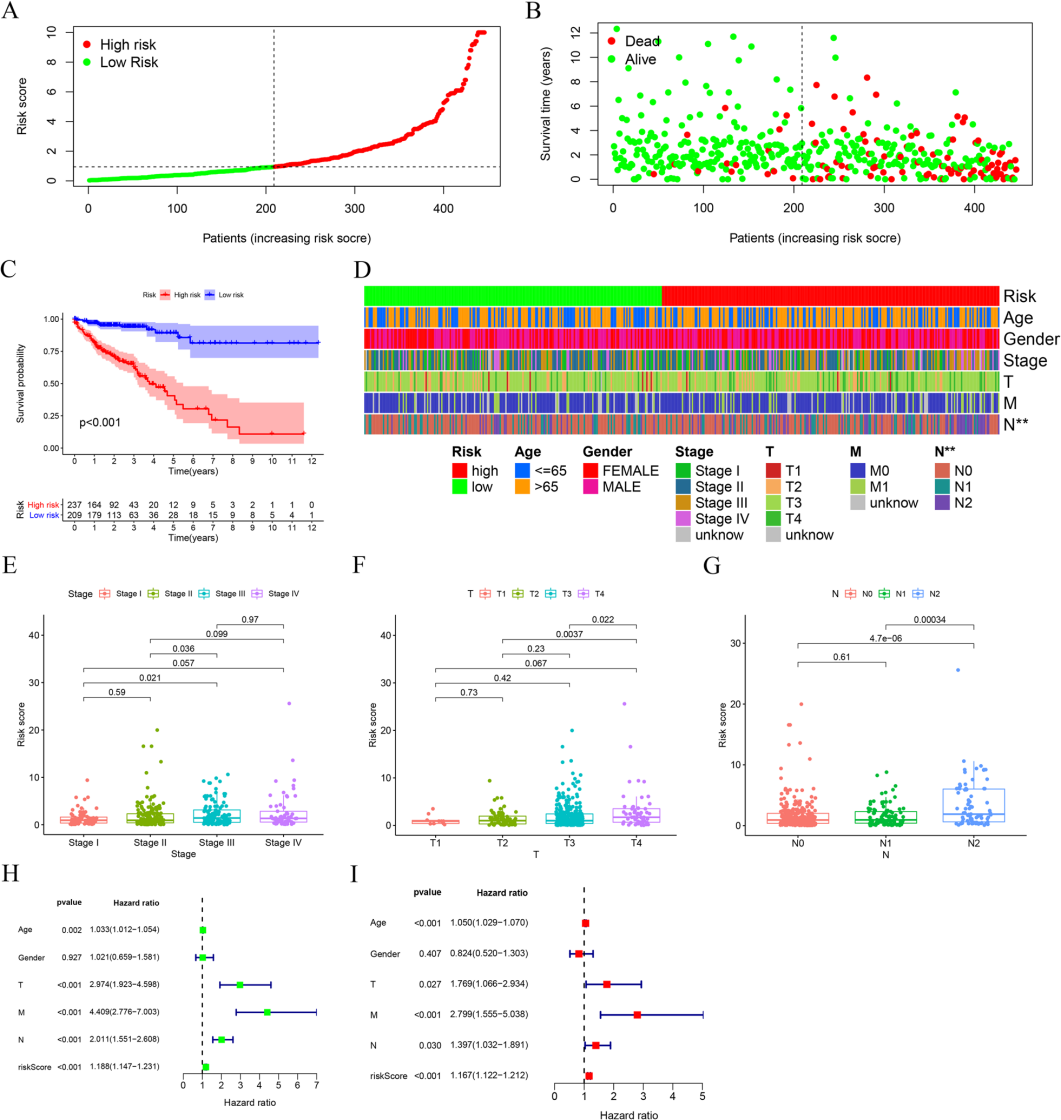
Survival predictability and clinical values of the risk model. **A** The risk plot shows the risk scores of individual patients in the high- and low-risk groups. **B** The scatter plot represents the survival status distribution of individual patients in the high- and low-risk groups. **C** Kaplan–Meier curves indicate patients in the low-risk group experiencing a longer survival time. **D**–**G** The strip chart (**D**) together with the box plots revealed that tumor grade (**E**), T stage (**F**), and N stage (**G**) were statistically significantly related to the risk score. **H** The univariate Cox regression analysis demonstrated that the T stage, N stage, M stage, and risk score were statistically different. **I** The multivariate Cox regression analyses indicated that the T stage, N stage, M stage, and risk score can serve as the independent prognostic predictor

The risk score of individual patients and their survival statuses were illustrated in Fig. 5A–B, from which we can recognize apparent differences in survival outcomes between the two subgroups of the patients. Concordantly, we conducted the Kaplan–Meier analysis and observed that the life expectancy of patients in the high-risk group was substantially decreased when compared to patients in the low-risk group (*P* value < 0.001) (Fig. 5C). To estimate the relationship between the risk model and the clinical traits, we charted a clinical-related heat map. Our findings suggested that the T grade and N grade were significantly different between the two groups (Fig. 5D). We next examined the risk score of patients across clinical traits including tumor stage, T grade, and N grade, respectively. We observed that the differences across tumor stages, T grades, as well as N grades were all of statistical significance (Fig. 5E–G). Finally, we conducted the univariate and multivariate cox analyses and found the pair-risk model can serve as an independent prognostic predictor (Fig. 5H and I).

Taken together, the irlncRNAs-based pair-risk assessment model not least can be utilized as a robust predictor of survival outcomes, but also serve as a reliable indicator of tumor aggressiveness of colon cancer.

### Assessment of the pair-risk model with tumor immune microenvironment

**Fig. 6:**
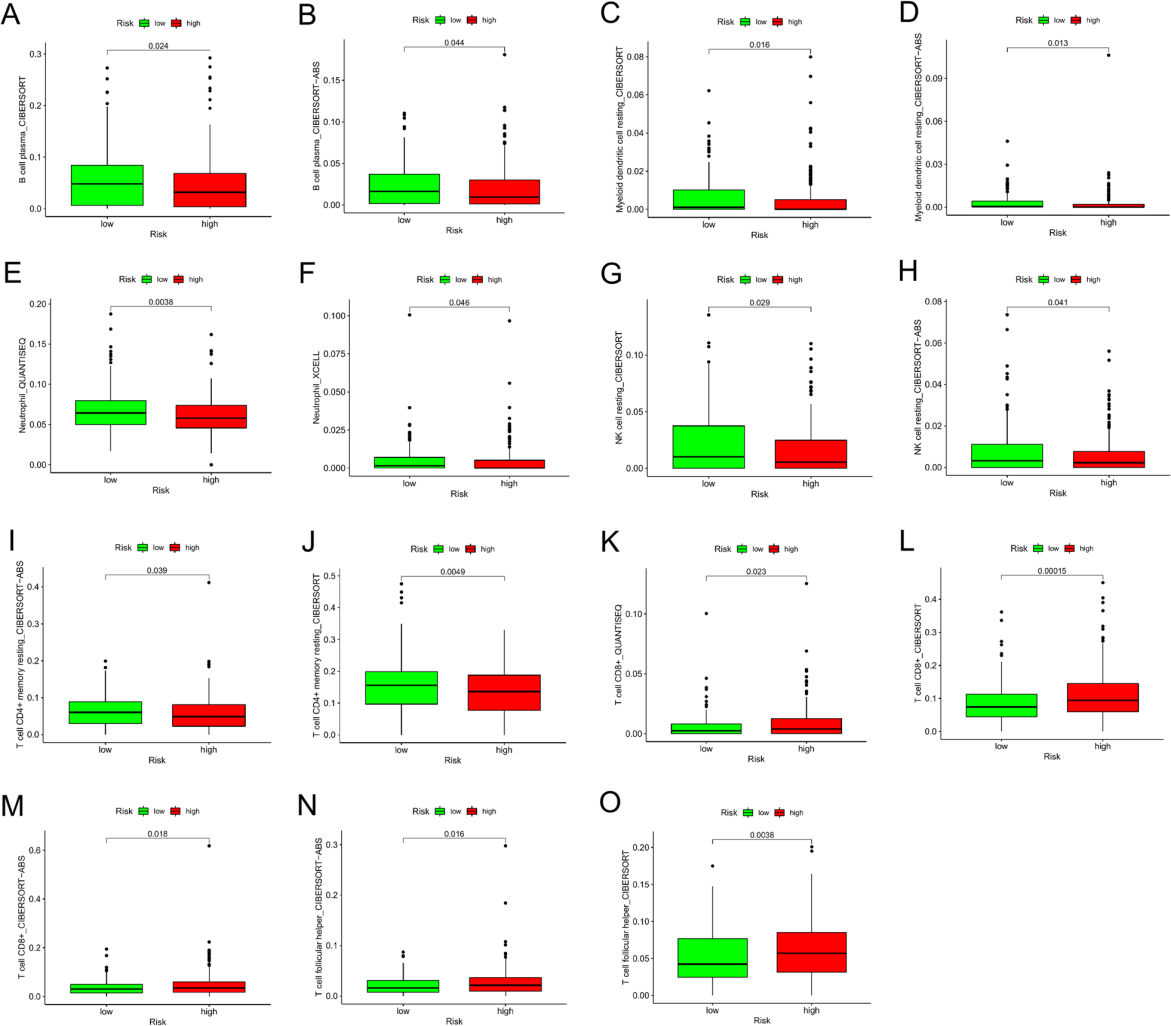
Investigation of tumor-related immune infiltrates by the pair-risk model. **A**–**O** The scatter chart and box plots revealed the relationship between the pair-risk model and the overlapped immune infiltrates by multiple algorithms

To develop a risk model to reflect the landscape of the immune microenvironment of patients with colon cancer, we first performed the correlation analysis of immune cells via 7 different algorithms, which are illustrated with distinct colors (Fig. 6, Additional file 4: Figure S1). Of note, the infiltrating patterns of B cells, dendritic cells, neutrophils, NK resting cells, CD4 + T memory cells, CD8 + T cells, T cell follicular helper were overlapped by more the two distinct algorithms, providing strong evidence for the risk model to predict the infiltration of certain types of immune cells (Fig. 6A–O).

### Estimation of the chemotherapeutic responsiveness of colon cancer by the pair-risk model

**Fig. 7:**
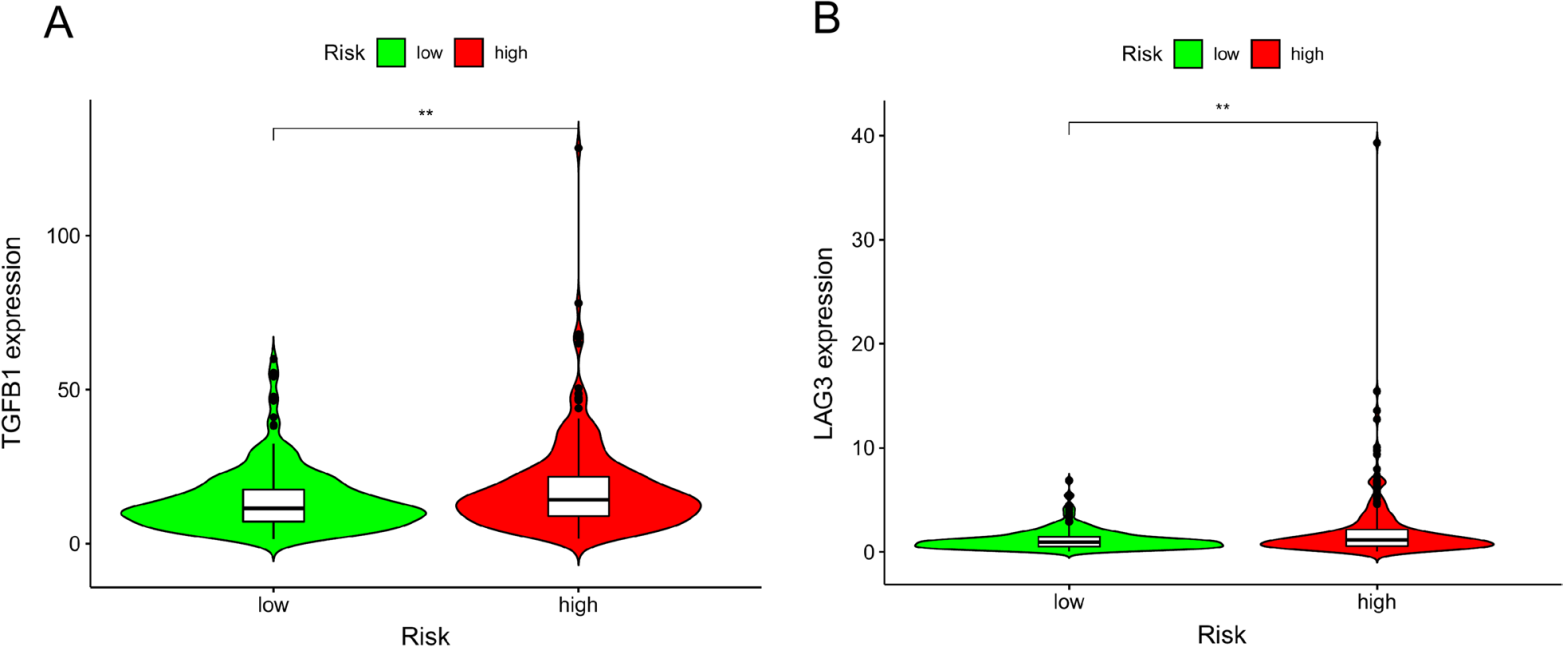
Evaluation of the relationship of the pair-risk model with certain immune-related genes. **A**, **B** The violin plots represent the relation of the pair-risk model with TGF-β and LAG3 expression

To investigate whether this risk model can be employed to predict the chemotherapeutic sensitivity for colon cancer patients, we examined the capacity of our pair-risk model for forecasting drug sensitivity by analyzing the immune-related gene expression as well as the differences of IC50 between the high- and low-risk groups. Our observations indicated that some immune-correlative genes, such as TGFB2 and LAG3, were shown a significantly positive correlation with risk score value (Fig. 7A and B). Moreover, we explored the relation of this model to the expression levels of other immune checkpoints like LAIR1, CD300A, TIGIT, LILRB1, KIR3DL1, HAVCR2, CD274 and CTLA4. Our observations revealed that these immune checkpoints tended to be upregulated in the high-risk group versus the low-risk group, although the differences did not reach statistical significance (Additional file 5: Figure S2).

**Fig. 8:**
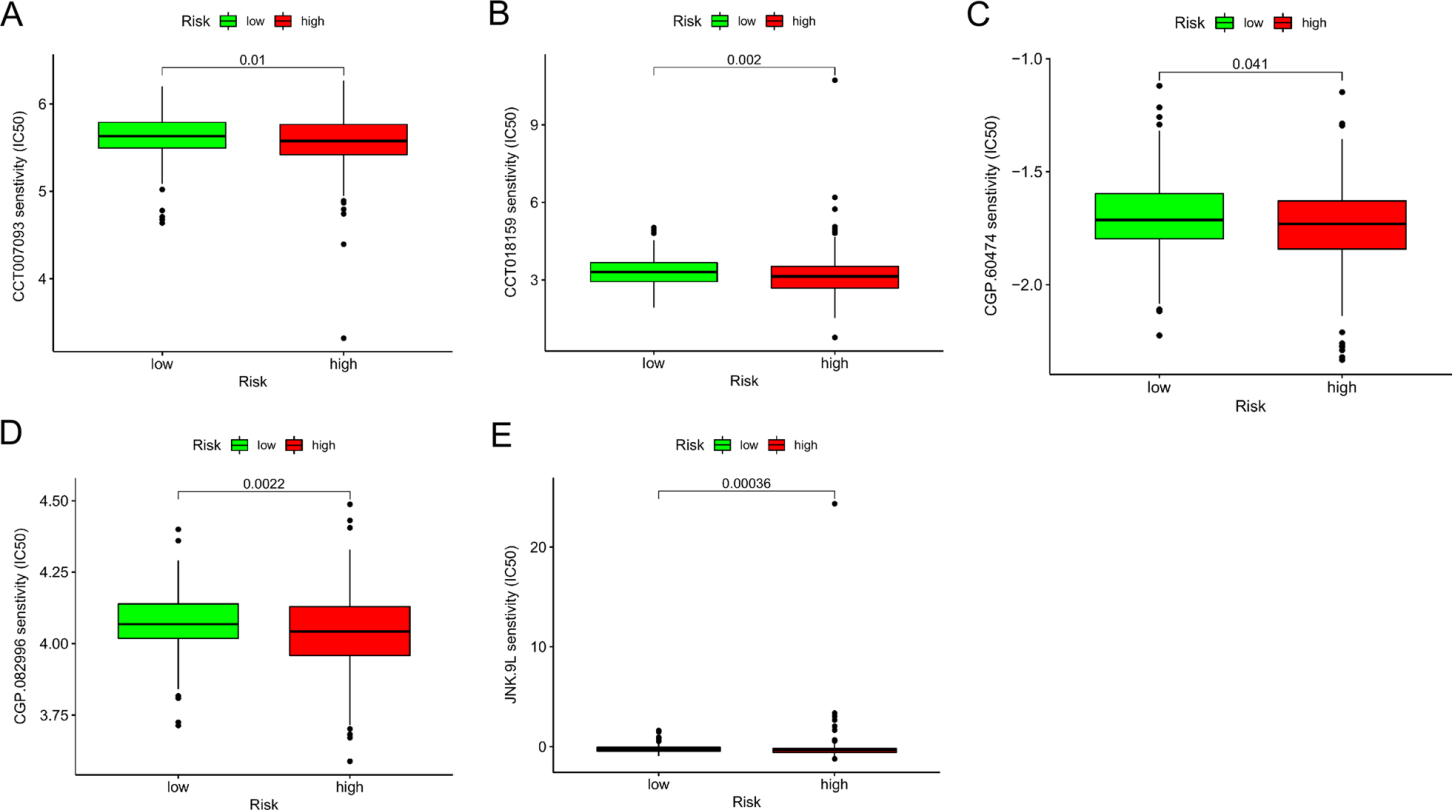
Evaluation of the relationship of the pair-risk model with certain immune-related genes and therapeutics. **A**–**E** The box plots reflect the association of the pair-risk model with certain therapeutics

Additionally, we identified 5 chemotherapeutic drugs, including CCT007093, CCT018159, CGP.60474, CGP.082996, JNK.9L, that appeared to have an inverse correlation between risk score and IC50 value (Fig. 8A–E), implicating that our risk model can serve as a tool to assist the chemotherapeutic medication.

### Construction and validation of an exp-risk model based on the selected core irlncRNAs

**Fig. 9:**
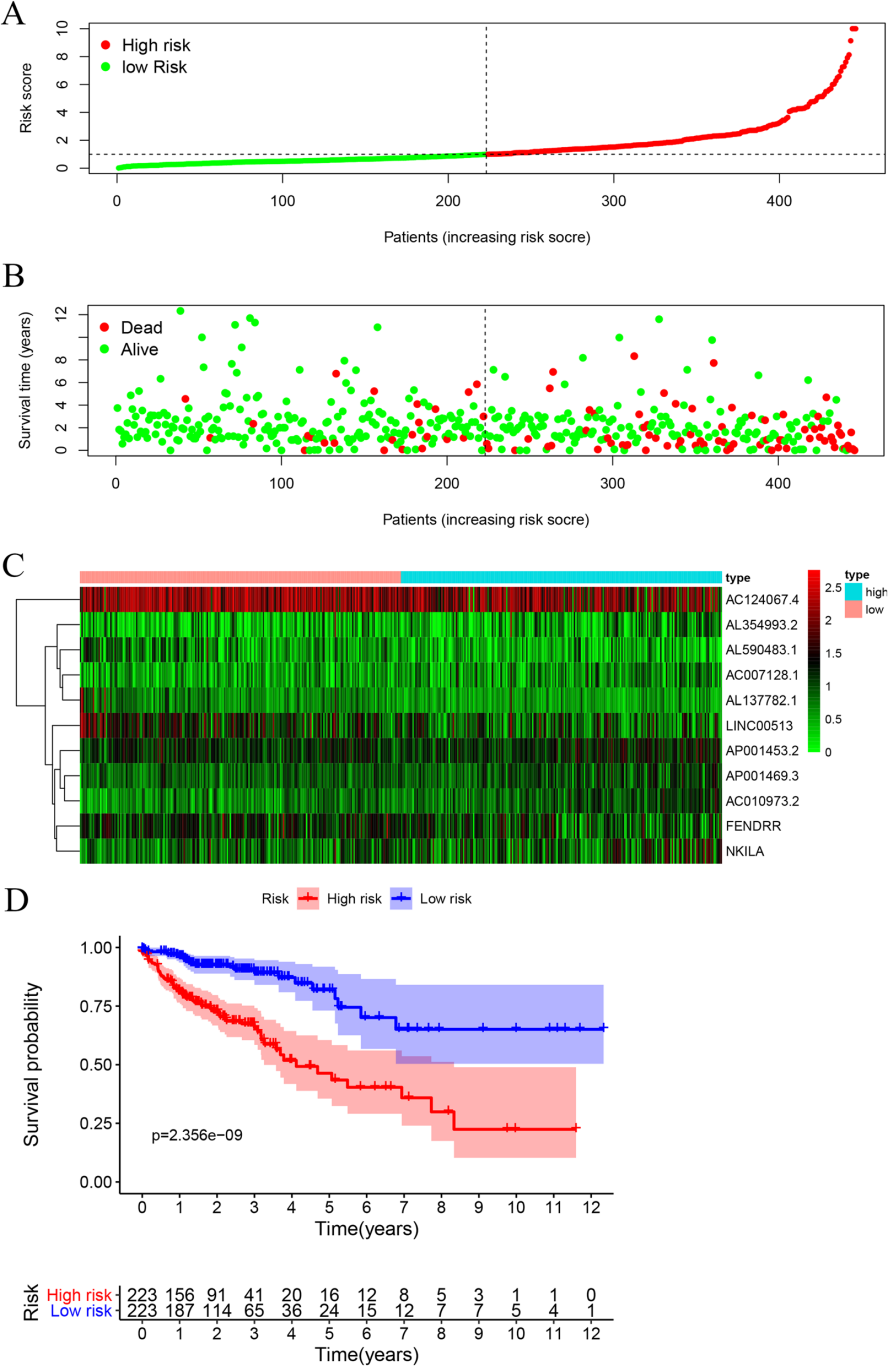
Illustration of the prognostic predictability of the exp-risk model. **A** The risk score distribution of individual colon cancer patients according to the exp-risk model. **B** The survival status of colon cancer patients involved based on the exp-risks core. **C** Expression heatmap of the eleven core irlncRNAs among the low-risk and high-risk groups. **D** Kaplan–Meier curve of the overall survival between the low-risk and high-risk groups

We next constructed an irlncRNA exp-risk model based on the 11 core irlncRNAs. Again, we divided the patients into a high-risk group and a low-risk group according to the median risk value of the expression risk model (Fig. 9A). Notably, patients in the high-risk group tended to have shorter life expectancy when compared to those in the low-risk group (Fig. 9B). Moreover, we illustrated the expression levels of the core lncRNAs involved in those risk groups (Fig. 9C). Additionally, we plotted the Kaplan–Meier curves comparing the survival outcomes of the groups and observed consistent results (Fig. 9D).

### Independent prognostic analysis of the exp-risk model

**Fig. 10:**
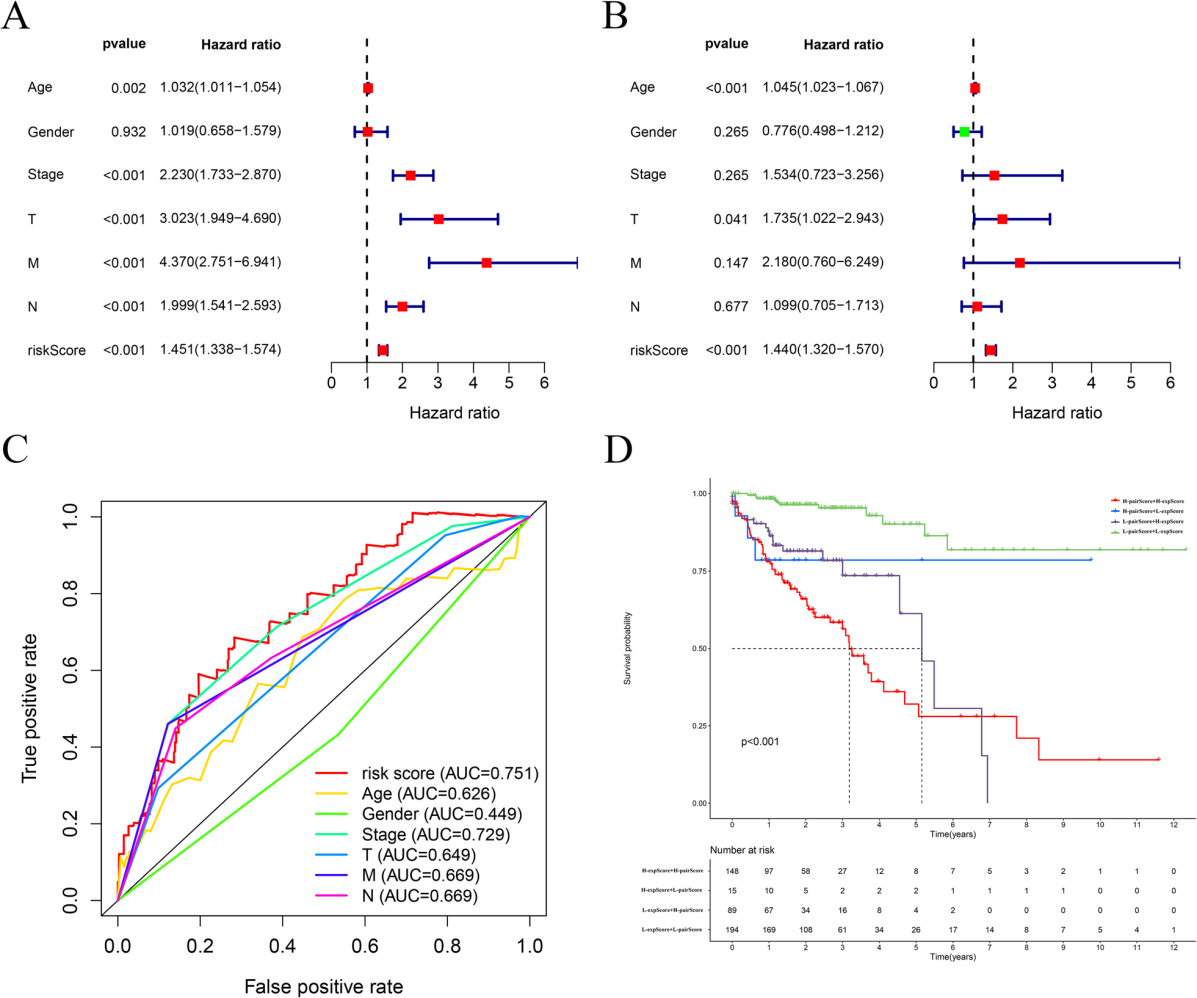
Evaluation of the relationship between the exp-risk model and clinical traits of colon cancer patients. **A** The univariate cox analysis revealed that age, stage, T, N, M, and exp-risk score can be regarded as risk factors. **B** The multiple cox analysis demonstrated that the exp-risk score remained as a risk factor when considering the whole characters. **C** The multiple ROC curves exhibited that the exp-risk score was the most optimal parameter to predict the prognosis of the colon cancer patients. **D** The evaluation of the exp-risk score and the pair-risk score shows robust predictability for the survival status of colon cancer patients

To evaluate whether this exp-risk model can be applied to predict prognostic outcomes of patients with colon cancer, we performed univariate independent prognostic analysis. Our observations suggested that the risk score, alongside tumor stage, T grade, N grade, and M grade, was significantly associated with survival outcomes of colon cancer patients (Fig. 10A). Moreover, we performed the multifactorial independent prognostic analysis and recognized that only the risk score could serve as an independent high-risk factor for predicting the prognosis of colon cancer (Fig. 10B). Then, we plotted the curve of the exp-risk model together with the curves of other clinic traits. By comparing the AUC of the exp-risk model to those of the clinical traits, we found the AUC value of the exp-risk model is 0.751, while the AUC values obtained from other clinical traits were less than 0.75, indicating that this model outperforms clinical traits for predicting the survival outcomes of colon cancer patients (Fig. 10C).

Additionally, to explore whether combining the pair-risk model with the exp-risk model could produce a more robust prognostic predictability, we categorized the patients involved into four groups: high pair score + high expression score; high pair score + low expression score; low pair score + high expression score; low pair score + low expression score. As expected, we found patients in the high pair score + high expression score group had the remarkably worst life expectancy, with a 5-year survival rate of only around 25% (Fig. 10D).

### GSEA enrichment analysis

**Fig. 11:**
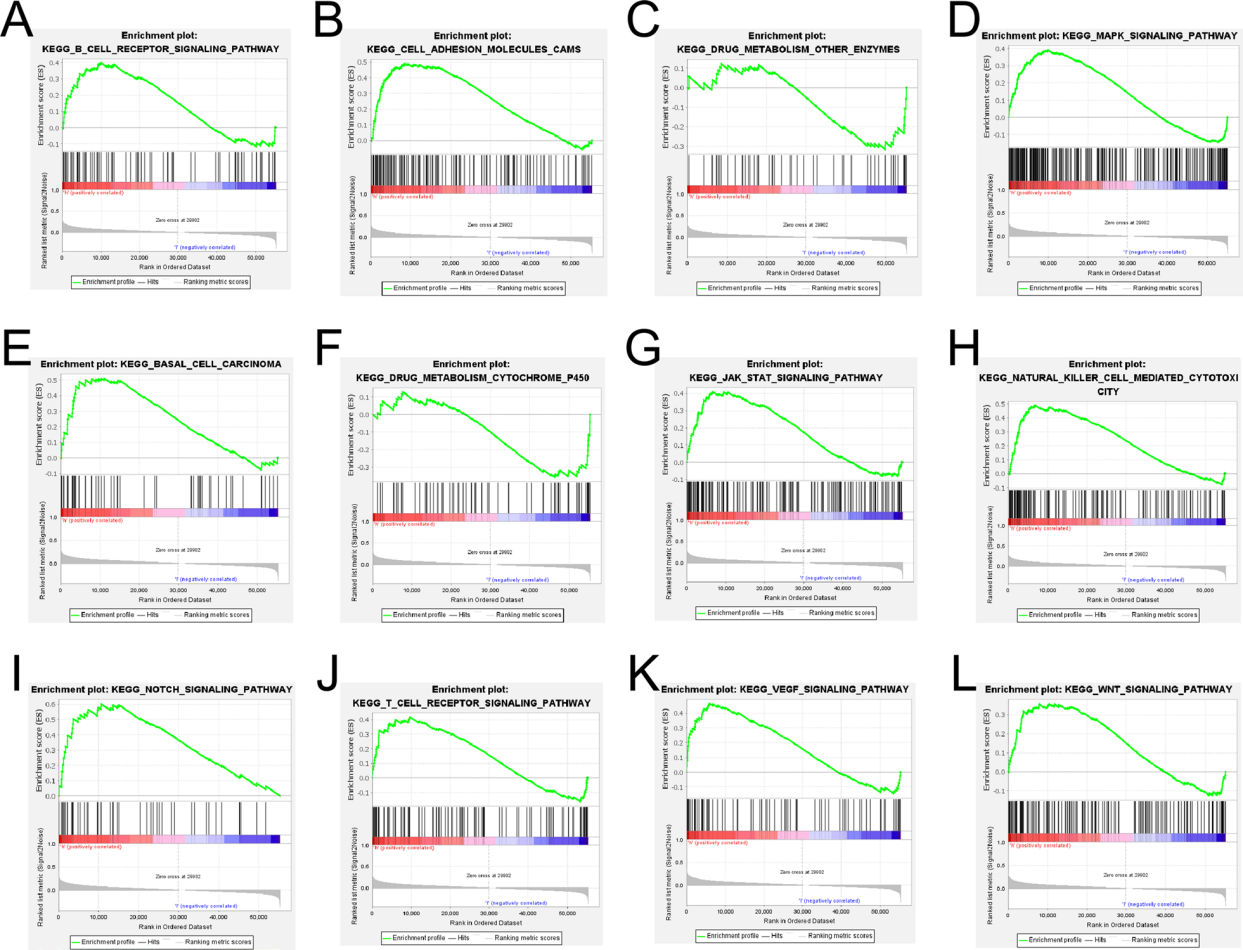
GSEA enrichment analysis according to the pair-risk model. **A**–**L** GSEA enrichment analysis of signal pathway based on the exp-risk model

**Fig. 12:**
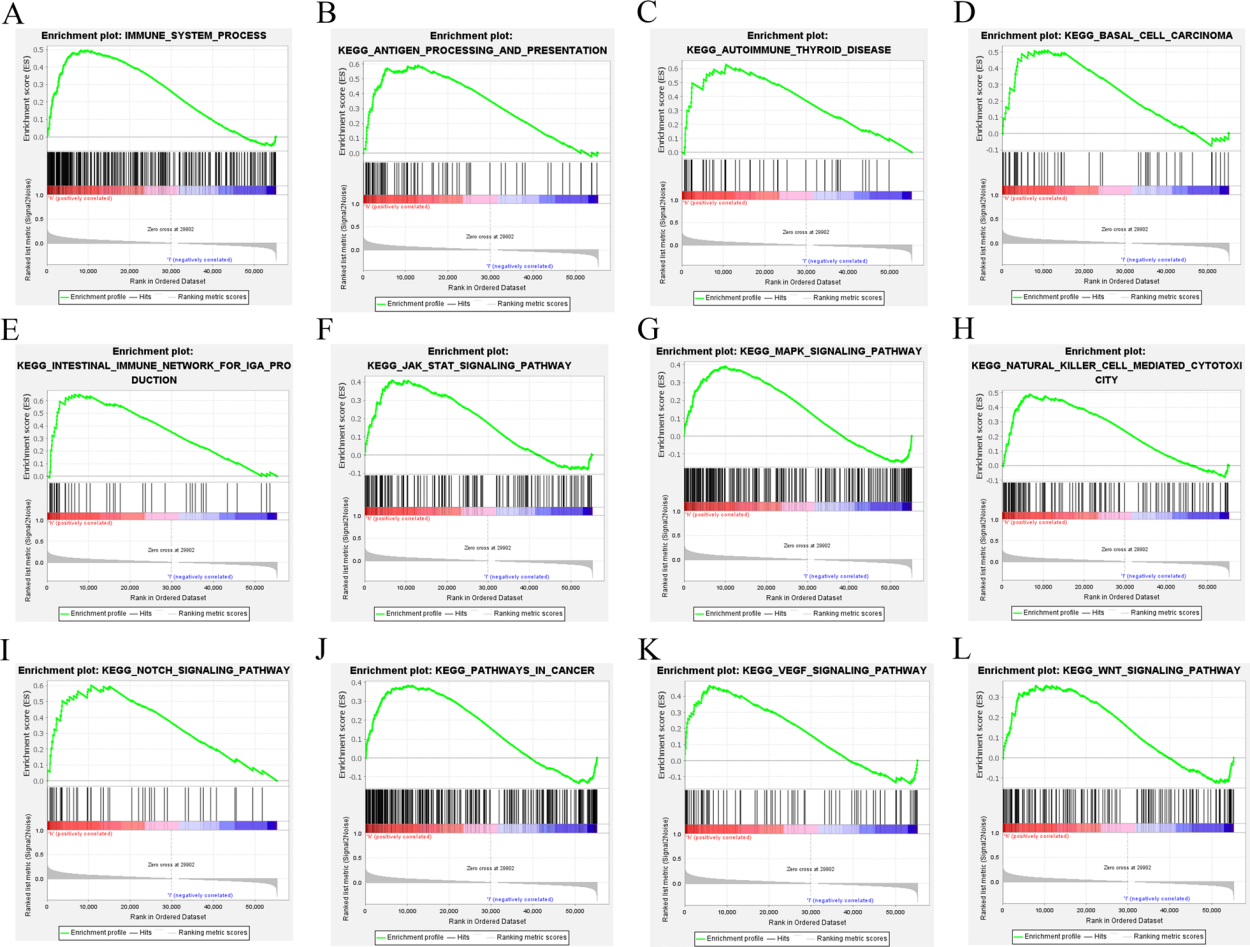
GSEA enrichment analysis according to the exp-risk model. **A**–**L** GSEA enrichment analysis of signal pathway based on the exp-risk model

Finally, we performed a GSEA enrichment analysis on both the pair-risk model and the exp-risk model. Our findings demonstrated that immune-related signaling and cancer-associated signaling pathways were significantly overactivated in the high-risk group of both of the two models (Figs. 11A–L; 12A–L). These results further confirmed the accuracy and reliability of both the pair-risk model and the exp-risk model.

## Discussion

The advent of high-throughput technologies and the evolution of conventional approaches have left a burst of non-coding RNA recognition and exploration. Accumulating studies have indicated that lncRNA participates in the development of malignant tumors via orchestrating the landscape of the tumor immune microenvironment [16]. Furthermore, a wealth of scientists has engaged in building up a model to predict the prognosis of cancer patients based on quantifying the expression levels of lncRNA. For example, Hong and colleagues constructed a risk assessment model based on the expression pattern of irlncRNA to predict the immune landscape and survival outcomes of hepatocellular carcinoma [17]. Zhong et al. investigated RNA sequencing profiles and identified a prognostic irlncRNA-scoring system and three molecular subtypes in clear cell renal cell carcinoma [18]. Similarly, Zhou et al. discovered an irlncRNA biomarker that can be utilized not least to predict prognostic outcomes of patients with diffuse large B cell lymphoma but can distinguish germinal center B-cell-like and activated B-cell-like subtypes [19]. However, due to the discrepancy of samples among different databases, the lncRNA-based risk assessment models mentioned above can hardly be transplanted to wider clinical practice. In the present study, we explored setting up a risk assessment model based on the irlncRNA pairing approach that circumvents the external interference among individual patients and, simultaneously, has the potential to foresee the aggressiveness, immune signature, and drug sensitivity of colon cancer. Growing evidence suggests that lncRNAs participate in a variety of immune-related biological activities [20, 21].

Our efforts provided additive evidence on the implication of irlncRNAs in the process of tumor evolution. Indeed, amassing studies have narrated the immune-related functions of some of the lncRNAs included in our risk model constructs. For example, FENDRR facilitates colorectal cancer growth and metastasis through interaction with miR-424-5p [22]. AC124067.4 was reported to affect the immunotherapy and prognosis outcomes of colon cancer patients via impacting genome instability [23]. lncRNA NKILA plays a crucial role in constraining tumor progression in a variety of cancer types, including colorectal cancer [24]. However, the role of several other lncRNAs involved in our risk models is not reported, which necessitates and navigates future studies to uncover their contribution to tumor progression.

It is well documented that the TME plays a crucial role in the development and progression of malignant carcinoma [25]. Immune infiltrates make up an essential part of the TME, exerting various functions that impact a series of bioactivities of cancer cells, such as promoting immune escape, accelerating tumor progression, and boosting drug resistance [26]. The composition of the TME is closely associated with the dynamics of tumorigenesis, malignant progression, and chemotherapeutic response [25, 27]. Intratumoral immune cells and fibroblasts constitute the major part of the TME and play crucial roles in determining the biological activities of cancer cells [28–30]. Therefore, improved knowledge of the architecture of tumor components, such as immune cells and fibroblasts, can help oncologists to choose a suitable therapeutic regimen to treat cancer patients [31]. To determine the relationship between risk scores and TME components, we used seven distinct computational methods. Our observations indicated that the pair-risk score was robustly indicative of the landscape of immune infiltration of colon cancer.

Additionally, we reused the 11 core lncRNAs to develop an exp-risk model, which also displayed a robust role in predicting the clinical and prognostic traits of colon cancer patients. Intriguingly, we found that the 5-year-survival AUC value in the pair-risk model is 0.843 and 0.751 in the exp-risk model, indicating the outperformance of the pair-risk model. Furthermore, our pair-risk model showed superior over the previously reported lncRNA-based risk-assessment model. For example, Wu et al. developed an irlncRNA-based model utilized to predict prognosis and therapeutic response in bladder cancer with a 5-year AUC value of merely 0.75 [32]. Lin et al. used 9 irlncRNAs to develop a risk model to predict the prognostic outcomes of colon cancer in the light of a 5-year AUC value of 0.78 [33].

Finally, we scrutinized the performance of this model to predict immune-related gene expression between high- and low-risk groups. Increased TGF-β expression and activation of the TGF-β receptor-initiated signaling pathway are observed in various cancers [34]. Patients with a higher level of TGF-β gene expression are associated with a worse prognosis [35]. Mechanically, TGF-β signaling facilitates tumor progression via inducing epithelial-to-mesenchymal transition and immune surveillance evasion [36, 37]. In the light of our model, the level of TGF-β was higher in the high-risk model compared to the low-risk model, implying the overactivation of the TGF-related pathway in the high-risk model. Proposed as the next immune checkpoint, LAG3 is expressed on multiple cell types including CD4 + and CD8 + T cells and plays crucial roles in T cell regulation and homeostasis [38]. As shown in Fig. 7, the level of LAG3 expression was higher in the high-risk group compared to the low-risk group, demonstrating the potential effect of LAG3 on shaping the tumor immune microenvironment. This finding is consistent with a recently published study demonstrating that the expression level of LAG3 in colorectal cancer is tightly associated with the levels of AC124067.4, AL137782.1 and AC010973.2 which are involved in our models [39]. These results indicate a potential functional link of those lncRNAs to LAG3 expression.

Finally, we examined our model to predict the drug susceptibility between high- and low-risk groups. Activation of JNK signaling has been confirmed to render chemotherapy resistance in a variety of malignancies, including hepatocellular carcinoma [40], gastric cancer [41], and pancreatic cancer [42]. In the setting of colon cancer, activating JNK signaling pathway contributes to 5-fluorouracil resistance in p53-defective or mutant colon cancer cells by inducing pro-survival autophagy [43]. Our findings suggest that a high score of irlncRNA pairs is positively associated with JNN inhibitor resistance. CCT007093 selectively and potently inhibits the activity of PPM1D, which overexpress on various cancers via activating p38 kinase activity [44]. CCT018159 works as an effective anticancer agent by inhibiting heat shock protein 90 (Hsp90) ATPase activity [45]. CGP.60474 and CGP.082996 are cyclin-dependent kinase-associated inhibitors and confer tumor cell death via modulating cell cycle arrest. Compensatory to previous investigations, this irlncRNA-based risk assessment model can serve as a chemotherapeutics-selecting tool to assist clinicians in determining suitable regimens.

Additionally, we reconstructed an exp-risk model by using the same pool of core irlncRNAs and found that the exp-risk model was also predictive of survival outcomes of patients with colon cancer with high performance. Importantly, the combination of the pair-risk model and exp-risk model can yield a more robust strategy to forecast the prognostic outcomes of colon cancer patients. Those results mean that the irlncRNAs involved might play a vital role in the progression of colon cancer, which further highlights the potential of studying those irlncRNAs to obtain deeper insight into the evolution of the disease.

However, the present study has some shortcomings and limitations. For example, the risk assessment models were constructed entirely based on the raw dataset from TCGA, but not validated by the clinical samples, implying the lack of clinical evidence. Additionally, this lncRNA pairs-based risk model can merely be acted as a biomarker to predict survival outcomes, tumor aggressiveness, and therapeutic resistance, whereas it failed to figure out the specific biological function of individual lncRNAs involved in this model.

## Conclusion

In conclusion, this study suggested a pair-risk assessment model, which was built based on 11 pairs of irlncRNAs. This risk model is not merely associated with the survival outcomes of the patients with colon cancer, but also linked to the tumor grade, tumor microenvironment, and chemotherapeutic resistance as well. We reused the core irlncRNAs involved to establish an exp-risk model, which is also indicative of survival outcomes of colon cancer patients. The identification of those models provides alternative measures to predict the biological characteristics of colon cancer and guide the treatment scheme.

## Supplementary Information

**Additional file 1: Table S1.** The risk model of immune lncRNA pairs.

**Additional file 2: Table S2.** Immune lncRNA pairs with survival significance.

**Additional file 3: Table S3.** The filtered immune lncRNAs.

**Additional file 4: Fig. S1.** Investigation of tumor-related immune infiltrates by the risk model. A-H. The scatter chart and box plots revealed the relationship between the pair-risk model and non-overlapped immune infiltrates predicted by individual algorithms. I. The overall immune infiltrating landscape is predicted by the 7 distinct algorithms.

**Additional file 5: Fig. S2.** Evaluation of the relationship of the pair-risk model with other immune-related genes. A-H. The violin plots chart the relationship between the pair-risk model and other immune checkpoint gene expressions.

## Data Availability

The datasets analyzed during the current study are available in TCGA (https://portal.gdc.cancer.gov/).

## Abbreviations

irlncRNA: Immune-related lncRNA
TCGA: The Cancer Genome Atlas
ROC: Receiver operating characteristic
AUC: Areas under the curve
lncRNA: Long non-coding RNA
TME: The tumor microenvironment
DEirlncRNA: Different expressed immune-related lncRNA
