# Supplementary material for: Construction of immune-related lncRNA signature to predict aggressiveness, immune landscape, and drug resistance of colon cancer
Source: BMC Gastroenterol. 2022 Mar 17;22:127. doi: 10.1186/s12876-022-02200-5 (PMC8928673; doi:10.1186/s12876-022-02200-5)
Supplement: Supplementary file 1 — Additional file 1: Table S1. The risk model of immune lncRNA pairs. [file 12876_2022_2200_MOESM1_ESM.docx]

Table S1 The critical DEirlncRNAs with apparent risk in colon cancer.

| ASMTL-AS1，AC105460.1，GABPB1-AS1，AP001469.3，LINC00941，AC011462.4，AP001453.2，AC006042.1，AL161729.4，AC087741.1，AP001628.1，AL031600.1，AC007128.1，MCM3AP-AS1，STAG3L5P-PVRIG2P-PILRB，AC245884.8，AC124067.4，LINC00513，AL445222.1，AL354836.1，ZKSCAN2-DT，AP001160.1，SNHG7，FENDRR，AC063948.1，AC008610.1，AL451050.2，LINC02381，AC004585.1，AC048344.4，AC074117.1，AC005837.3，AC008735.2，MMP25-AS1，AC073957.3，AL137782.1，AP006621.2，AC027796.4，NKILA，AC127024.4，AC048341.2，AC132872.3，AC022144.1，AC008760.1，LINC00174，AL118505.1  AL590483.1，BX470102.1，SNHG16，PTOV1-AS2，AC018653.3，AC010973.2，LINC01138，AL354993.2，LENG8-AS1 |
| --- |
