# Supplementary material for: Construction of immune-related lncRNA signature to predict aggressiveness, immune landscape, and drug resistance of colon cancer
Source: BMC Gastroenterol. 2022 Mar 17;22:127. doi: 10.1186/s12876-022-02200-5 (PMC8928673; doi:10.1186/s12876-022-02200-5)
Supplement: Supplementary file 2 — Additional file 2: Table S2. Immune lncRNA pairs with survival significance. [file 12876_2022_2200_MOESM2_ESM.docx]

Table S2 Immune lncRNA pairs with survival significance.

| Gene | HR | HR.95L | HR.95H | pvalue |
| --- | --- | --- | --- | --- |
| AC105460.1\|AL590483.1 | 1.922011912 | 1.258529722 | 2.935274175 | 0.002491854 |
| GABPB1-AS1\|LINC00513 | 2.178243136 | 1.45283578 | 3.265849607 | 0.000164848 |
| AP001469.3\|LINC00513 | 1.791526488 | 1.194073524 | 2.687914181 | 0.004849587 |
| AP001469.3\|FENDRR | 1.957294255 | 1.30442651 | 2.936923446 | 0.001180417 |
| AP001469.3\|AL137782.1 | 4.067780542 | 1.849923431 | 8.944607255 | 0.000482875 |
| LINC00941\|AL590483.1 | 1.85322286 | 1.233962644 | 2.783256839 | 0.002947928 |
| AC011462.4\|LINC00513 | 1.827821416 | 1.213316453 | 2.753552975 | 0.003916437 |
| AC011462.4\|FENDRR | 1.908385858 | 1.262133101 | 2.885540822 | 0.002187219 |
| AC011462.4\|AL137782.1 | 2.530035776 | 1.540246233 | 4.155881633 | 0.000246567 |
| AP001453.2\|AC027796.4 | 0.503184374 | 0.314893085 | 0.804065019 | 0.004080848 |
| AL161729.4\|LINC00513 | 2.123127347 | 1.390840299 | 3.240968596 | 0.000485461 |
| AL161729.4\|FENDRR | 1.773461417 | 1.15984501 | 2.711711797 | 0.008184023 |
| AL161729.4\|AL137782.1 | 1.934258631 | 1.271049157 | 2.943518298 | 0.002073308 |
| AC087741.1\|LINC00513 | 2.046945646 | 1.358208946 | 3.084935122 | 0.000619534 |
| AP001628.1\|LINC00513 | 2.144962165 | 1.419883867 | 3.240309152 | 0.000288395 |
| AP001628.1\|FENDRR | 1.803197438 | 1.181607843 | 2.751776759 | 0.006261617 |
| AP001628.1\|AL137782.1 | 1.748407174 | 1.147456477 | 2.664090277 | 0.009320269 |
| AL031600.1\|LINC00513 | 1.872306067 | 1.246288309 | 2.812776133 | 0.002525922 |
| AL031600.1\|AL590483.1 | 1.988926435 | 1.24956161 | 3.165772965 | 0.00373842 |
| AC007128.1\|AL590483.1 | 2.168081672 | 1.410482759 | 3.332602334 | 0.00041879 |
| MCM3AP-AS1\|AC245884.8 | 0.466253659 | 0.274756397 | 0.791218972 | 0.004685947 |
| MCM3AP-AS1\|LINC00513 | 2.005734942 | 1.308479183 | 3.07454082 | 0.001404819 |
| MCM3AP-AS1\|AL137782.1 | 1.971093289 | 1.269713709 | 3.059909273 | 0.002493386 |
| STAG3L5P-PVRIG2P-PILRB\|LINC00513 | 1.923950881 | 1.26661287 | 2.922429638 | 0.002154398 |
| STAG3L5P-PVRIG2P-PILRB\|AL137782.1 | 2.071785553 | 1.313139506 | 3.268727625 | 0.001742716 |
| STAG3L5P-PVRIG2P-PILRB\|AL590483.1 | 1.911093189 | 1.194034274 | 3.058770804 | 0.006955749 |
| AC245884.8\|LINC00513 | 1.928953283 | 1.28692504 | 2.891280107 | 0.001464773 |
| AC245884.8\|AL137782.1 | 3.011093891 | 1.659509957 | 5.463472143 | 0.000287522 |
| AC124067.4\|SNHG7 | 0.528627558 | 0.343620493 | 0.813243391 | 0.003724552 |
| LINC00513\|ZKSCAN2-DT | 0.492889314 | 0.329375204 | 0.737577915 | 0.000581728 |
| LINC00513\|AP001160.1 | 0.468682104 | 0.311948973 | 0.704162967 | 0.000263598 |
| LINC00513\|AC063948.1 | 0.441974039 | 0.289585914 | 0.674553014 | 0.000153673 |
| LINC00513\|AC008610.1 | 0.542177079 | 0.347428476 | 0.846090649 | 0.007017509 |
| LINC00513\|AL451050.2 | 0.378101522 | 0.251242316 | 0.569015457 | 3.11E-06 |
| LINC00513\|LINC02381 | 0.441622948 | 0.292942046 | 0.665765911 | 9.52E-05 |
| LINC00513\|MMP25-AS1 | 0.473742529 | 0.315277854 | 0.711854579 | 0.000323312 |
| LINC00513\|AC073957.3 | 0.560230322 | 0.37196602 | 0.843781413 | 0.005556388 |
| LINC00513\|AP006621.2 | 0.44004576 | 0.290847472 | 0.665779453 | 0.000102138 |
| LINC00513\|AC027796.4 | 0.470200585 | 0.31365474 | 0.704878844 | 0.000259186 |
| LINC00513\|NKILA | 0.495331657 | 0.330660265 | 0.742010685 | 0.000656589 |
| LINC00513\|AC048341.2 | 0.503074726 | 0.333142121 | 0.759688325 | 0.001087218 |
| LINC00513\|AC022144.1 | 0.504766935 | 0.331242107 | 0.769194656 | 0.001468176 |
| LINC00513\|AC008760.1 | 0.493909026 | 0.325467913 | 0.749524349 | 0.000917049 |
| LINC00513\|LINC00174 | 0.497839048 | 0.330414525 | 0.750099342 | 0.000853593 |
| LINC00513\|AC010973.2 | 0.409140512 | 0.272890003 | 0.613419168 | 1.52E-05 |
| LINC00513\|LINC01138 | 0.456671424 | 0.304518429 | 0.684847845 | 0.000150093 |
| LINC00513\|LENG8-AS1 | 0.369087414 | 0.214951893 | 0.633748867 | 0.000302067 |
| AL445222.1\|AL137782.1 | 2.711799034 | 1.736224079 | 4.235544299 | 1.16E-05 |
| AL445222.1\|AL590483.1 | 1.754550181 | 1.152536573 | 2.671018354 | 0.008739841 |
| ZKSCAN2-DT\|AL590483.1 | 2.461770296 | 1.365867222 | 4.436970808 | 0.002723661 |
| AP001160.1\|AL137782.1 | 1.842797415 | 1.158425052 | 2.931482105 | 0.009855421 |
| FENDRR\|AC004585.1 | 0.525312037 | 0.348587793 | 0.791630522 | 0.002093227 |
| FENDRR\|AC005837.3 | 0.522371011 | 0.346741636 | 0.786959062 | 0.001897574 |
| FENDRR\|AC073957.3 | 0.559340998 | 0.372894131 | 0.839011199 | 0.00497792 |
| FENDRR\|AC027796.4 | 0.542397062 | 0.361312362 | 0.814238878 | 0.003163389 |
| FENDRR\|NKILA | 0.545335675 | 0.364238071 | 0.816474231 | 0.003233488 |
| FENDRR\|AC127024.4 | 0.453336413 | 0.301991711 | 0.68052829 | 0.000135125 |
| FENDRR\|AC132872.3 | 0.458801465 | 0.294577767 | 0.714577975 | 0.000567778 |
| FENDRR\|AC022144.1 | 0.526379218 | 0.34650383 | 0.799630646 | 0.002628796 |
| FENDRR\|AC008760.1 | 0.562305776 | 0.373434082 | 0.846703077 | 0.005837012 |
| FENDRR\|LINC00174 | 0.583552292 | 0.389358374 | 0.874601139 | 0.009081485 |
| FENDRR\|AC010973.2 | 0.547071764 | 0.363203278 | 0.824022065 | 0.003900322 |
| FENDRR\|LINC01138 | 0.474999579 | 0.314504956 | 0.717396007 | 0.00040202 |
| FENDRR\|LENG8-AS1 | 0.471642036 | 0.2839074 | 0.783516774 | 0.003707721 |
| AL451050.2\|AL137782.1 | 2.763179084 | 1.760214436 | 4.337629833 | 9.98E-06 |
| AL451050.2\|AL590483.1 | 2.400895919 | 1.53332705 | 3.759342284 | 0.00012903 |
| AL137782.1\|AP006621.2 | 0.449344817 | 0.260746775 | 0.774355748 | 0.003965446 |
| AL137782.1\|AC027796.4 | 0.392966205 | 0.225757986 | 0.684017611 | 0.000956887 |
| AL137782.1\|AC127024.4 | 0.472287553 | 0.286680082 | 0.778064286 | 0.00322757 |
| AL137782.1\|AC010973.2 | 0.435322922 | 0.253850887 | 0.746525049 | 0.002508815 |
| AL137782.1\|AL354993.2 | 0.573393441 | 0.383019828 | 0.85838908 | 0.006898465 |
| AP006621.2\|AL590483.1 | 2.424570309 | 1.367087233 | 4.300048337 | 0.002449135 |
| AL590483.1\|AC010973.2 | 0.438746184 | 0.254248355 | 0.757126685 | 0.00308223 |
| AL590483.1\|LINC01138 | 0.517041711 | 0.314635683 | 0.849656112 | 0.009245367 |
| AL590483.1\|AL354993.2 | 0.528254969 | 0.34865295 | 0.800375595 | 0.002609555 |
