# Supplementary material for: Construction of immune-related lncRNA signature to predict aggressiveness, immune landscape, and drug resistance of colon cancer
Source: BMC Gastroenterol. 2022 Mar 17;22:127. doi: 10.1186/s12876-022-02200-5 (PMC8928673; doi:10.1186/s12876-022-02200-5)
Supplement: Supplementary file 3 — Additional file 3: Table S3. The filtered immune lncRNAs. [file 12876_2022_2200_MOESM3_ESM.docx]

Table S3 The filtered immune lncRNAs.

| DEirlncRNA | RISKirlncRNA | COREirlncRNA |
| --- | --- | --- |
| AC254629.1  LINC01748  AC005392.2  AC010834.3  AP000254.1  MACC1-AS1  ZDHHC20-IT1  AL021707.6  AC018521.6  MBNL1-AS1  AC100814.1  AP002336.2  AP001554.1  CYTOR  BLACAT1  AF117829.1  ACBD3-AS1  INE1  AL033519.3  AL022322.1  AC090739.1  ARHGEF38-IT1  AC099518.2  AC108865.2  AP003419.3  LINC01705  AC008649.2  AC123023.1  NCBP2-AS1  AC021218.1  AC106820.3  AC253536.6  AC005256.1  LINC00106  MIR3142HG  AL117332.1  AC010536.2  AC004908.2  AL021578.1  LINC01473  AC145207.8  AC092171.4  AL135905.1  DLEU1  LINC01315  AP001429.1  AP001042.1  PVT1  MCF2L-AS1  AC010719.1  AC005261.1  AL354696.1  GAS5  AL356299.2  AC062037.2  AC108134.3  AC113143.1  AC103591.3  AL031673.1  LINC01232  AL133330.1  AL117379.1  AC243967.2  LINC00265  AL139349.1  AC027228.2  AL138689.1  AC020907.4  THUMPD3-AS1  CD44-AS1  AC020978.3  MIR181A2HG  AL121601.1  AL162724.2  AC005519.1  AL606834.1  SCARNA9  AP005271.1  LINC02441  AC011676.1  AL035071.2  SNHG15  AC233728.1  Z83843.1  AP005899.1  LMO7-AS1  AC124283.2  SP2-AS1  MIRLET7BHG  AP003352.1  AL161891.1  AC120498.10  AC068790.5  AC121761.1  AC092338.1  SNHG20  AL355488.1  AC004232.2  AP003390.1  AC092119.2  LINC01389  LINC00659  TUSC8  VPS9D1-AS1  AC079907.1  AC002128.2  AC016831.4  ZFAS1  AL138963.1  MAFG-DT  AC015922.3  AC005041.3  FTX  AL049539.1  AC007637.1  AL513327.1  AC104958.2  LINC01355  SLC12A9-AS1  AC016888.1  MIR17HG  LINC02362  AC104823.1  AC091057.1  AC004241.3  LINC01876  AGAP2-AS1  MIR222HG  AL031716.1  AC093585.1  AC007608.2  AC022211.1  AC145285.6  OGFRP1  ANKRD10-IT1  AC000061.1  AC009065.2  SNHG25  AC055717.2  LINC01558  B4GALT1-AS1  AC008555.6  AC079684.1  AC084125.4  AC058791.1  AL109614.1  AC130456.3  AC004837.2  AC093788.1  AC099850.3  HM13-IT1  AC008115.3  AC009269.5  AL117382.1  AC004264.1  AC011468.1  AC114296.1  AC004253.1  AC093732.1  AC136475.3  AL031275.1  SATB2-AS1  LINC01614  SNHG1  AC087222.1  SNHG12  AC092112.1  AP002387.1  AL121895.2  AC004492.1  AP000692.1  NALT1  AL035461.2  ARHGAP27P1-BPTFP1-KPNA2P3  TSPOAP1-AS1  AC015813.1  AC026356.1  N4BP2L2-IT2  LINC02487  MHENCR  GAS6-AS1  AC067930.4  MNX1-AS1  AC019080.5  AL133520.1  AC015922.2  AC007938.3  SNHG17  AL161772.1  AL121832.3  USP30-AS1  ABALON  AC108058.1  AC018809.1  AC008870.2  DIO3OS  AC112496.1  AL034550.1  EIF1AX-AS1  RHPN1-AS1  AC010280.1  AC131009.3  AC000123.1  AL442067.1  AL359881.1  AL596223.2  AL355075.2  PRR7-AS1  AL390726.4  LINC02195  AL035071.1  TFAP2A-AS1  AC092168.2  AP000866.6  UNC5B-AS1  AP000786.1  SERTAD4-AS1  AC125807.2  MIR4435-2HG  SNHG11  LINC02418  AC103706.1  LINC01871  NEAT1  AC009509.1  AC010542.5  AL353804.2  CASC19  AC010998.3  AC080129.2  AC132192.2  AC010761.1  RUSC1-AS1  AP002907.1  AL390719.2  LINC02577  MALAT1  AL157838.1  AC137630.3  AC015849.3  AL357079.1  LINC01811  AC007038.1  SNHG22  SNHG4  ALG13-AS1  AC131971.1  Z82243.1  AC115522.1  ATP2A1-AS1  AL353796.1  LINC01752  AC084117.1  AC020765.2  AL049840.2  AL136115.2  AL590723.1  Z68871.1  AL121832.2  AC136475.8  MIR100HG  AC090152.1  LINC01235  AL080317.1  AC087277.2  AP006621.4  LINCR-0001  AL031186.1  AC109322.1  AL078587.1  CDKN2B-AS1  AC092723.1  AC093620.1  LINC02595  AC009032.1  AL928654.2  ZNF433-AS1  GK-AS1  AC124319.1  AC127024.5  PSPC1-AS2  UBE2R2-AS1  AC016394.1  FMR1-IT1  AL031985.3  AC141002.1  AC090116.1  AL133410.1  LINC00114  B3GALT5-AS1  AL158837.1  WNT5A-AS1  AC007342.5  MIR22HG  AL442125.2  LINC00460  AL133243.2  AC007688.2  AC020656.2  GAPLINC  CASC9  LINC01082  AC007099.1  AL355472.1  LINC01311  AC018695.4  AL031670.1  AC073487.1  AC108727.1  PLAC4  LINC02163  AC009065.5  TMEM147-AS1  GK-IT1  DLEU2  LINC-PINT  AL163953.1  PAN3-AS1  AC104695.3  TRIM31-AS1  AC026368.1  SNHG3 | AC009237.15  AL162274.2  RAD51-AS1  AL513550.1  AC068580.3  AC002467.1  AP006621.3  AC007996.1  APTR  AC132872.1  ZEB1-AS1  AC069281.2  AC009118.3  AC104794.3  AC005253.1  AL139246.5  AC147067.1  AC004148.1  AL512413.1  AC102953.2  AP001505.1  LINC01503  AC107375.1  AC011472.1  AC245060.2  AC040977.1  CD27-AS1  AL162586.1  AL033384.2  KMT2E-AS1  AC138207.5  AC080112.1  AP001830.1  AC005261.3  AL022328.1  LINC01278  AL360181.2  AL133367.1  AC020558.2  DICER1-AS1  PPP1R26-AS1  LINC01089  AC068580.1  MIR210HG  AC093673.1  AC073896.3  AL122010.1  AL136295.6  AC090517.2  NIFK-AS1  AL022328.2  TOLLIP-AS1  AC026471.4  AC018647.2  AC015871.3  AC002128.1  ARRDC1-AS1  AL138756.1  AC084018.1  AC005332.4 | ASMTL-AS1  AC105460.1  GABPB1-AS1  AP001469.3  LINC00941  AC011462.4  AP001453.2  AC006042.1  AL161729.4  AC087741.1  AP001628.1  AL031600.1  AC007128.1  MCM3AP-AS1  STAG3L5P-PVRIG2P-PILRB  AC245884.8  AC124067.4  LINC00513  AL445222.1  AL354836.1  ZKSCAN2-DT  AP001160.1  SNHG7  FENDRR  AC063948.1  AC008610.1  AL451050.2  LINC02381  AC004585.1  AC048344.4  AC074117.1  AC005837.3  AC008735.2  MMP25-AS1  AC073957.3  AL137782.1  AP006621.2  AC027796.4  NKILA  AC127024.4  AC048341.2  AC132872.3  AC022144.1  AC008760.1  LINC00174  AL118505.1  AL590483.1  BX470102.1  SNHG16  PTOV1-AS2  AC018653.3  AC010973.2  LINC01138  AL354993.2  LENG8-AS1 |
