# Supplementary figures and images for: Construction of immune-related lncRNA signature to predict aggressiveness, immune landscape, and drug resistance of colon cancer
Source: BMC Gastroenterol. 2022 Mar 17;22:127. doi: 10.1186/s12876-022-02200-5 (PMC8928673; doi:10.1186/s12876-022-02200-5)

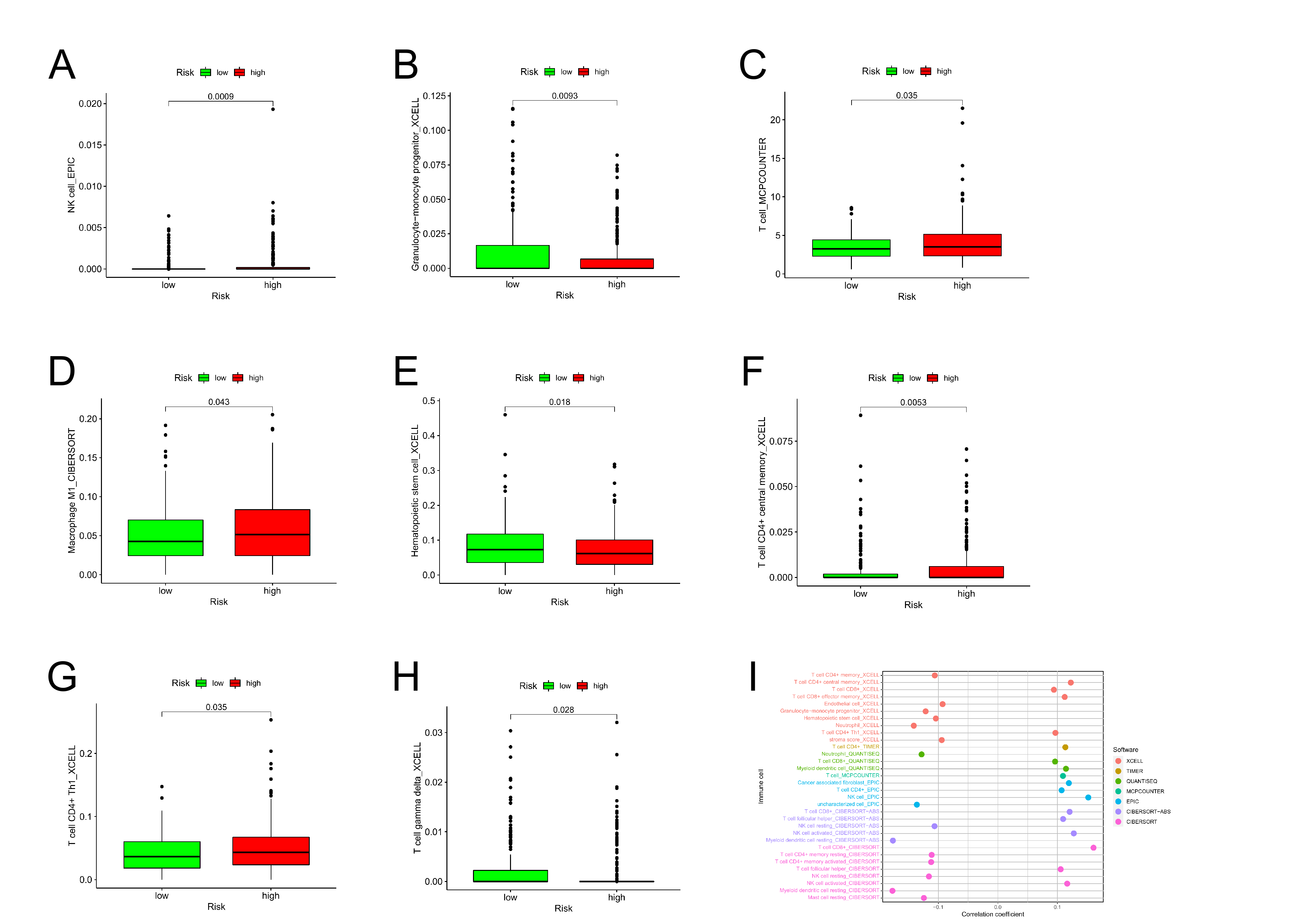

Supplement: Supplementary file 4 — Additional file 4: Fig. S1. Investigation of tumor-related immune infiltrates by the risk model. A-H. The scatter chart and box plots revealed the relationship between the pair-risk model and non-overlapped immune infiltrates predicted by individual algorithms. I. The overall immune infiltrating landscape is predicted by the 7 distinct algorithms. [file 12876_2022_2200_MOESM4_ESM.tiff]

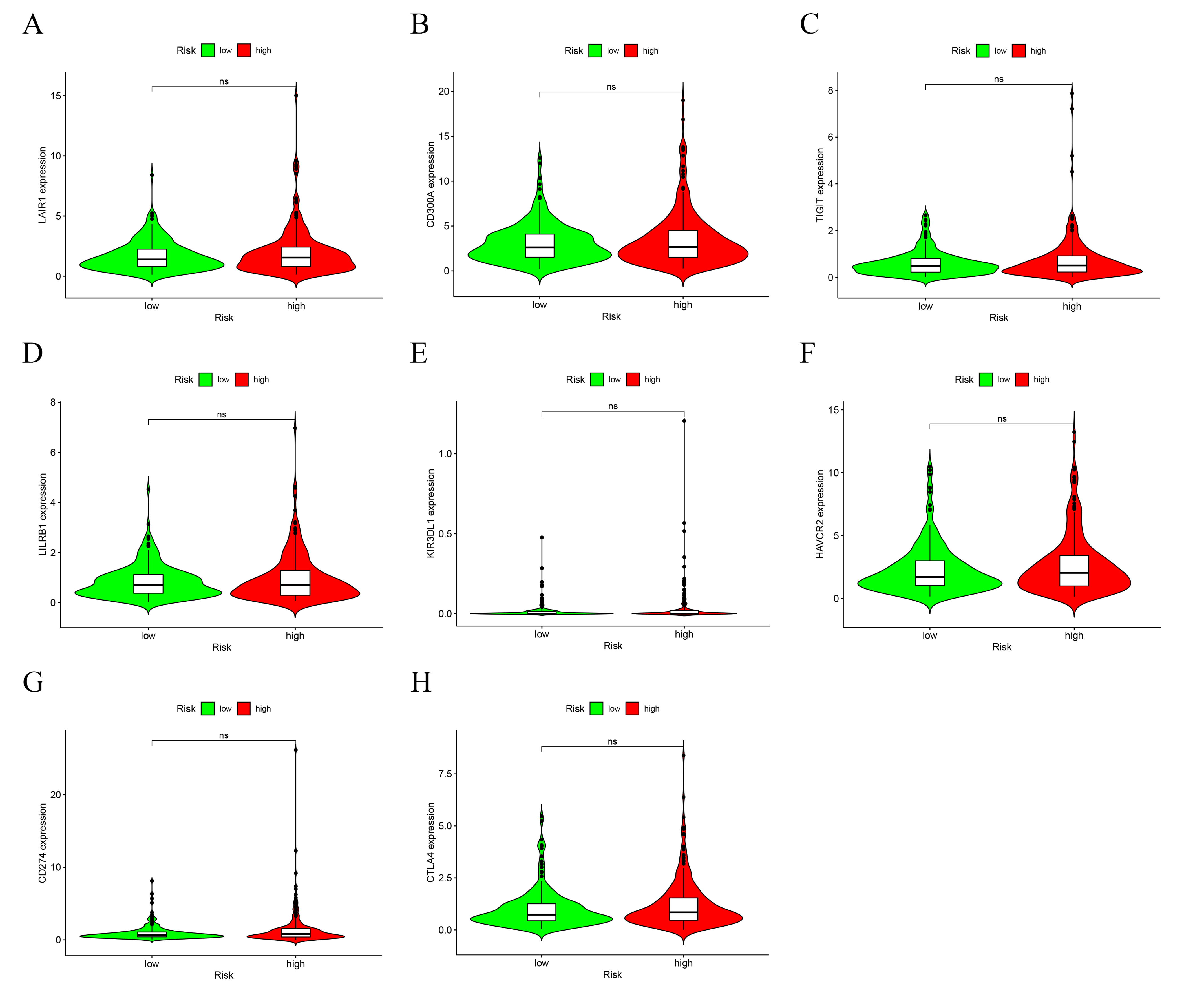

Supplement: Supplementary file 5 — Additional file 5: Fig. S2. Evaluation of the relationship of the pair-risk model with other immune-related genes. A-H. The violin plots chart the relationship between the pair-risk model and other immune checkpoint gene expressions. [file 12876_2022_2200_MOESM5_ESM.tif]
